# Supplementary material for: Comprehensive Annotation and Functional Exploration of MicroRNAs in Lettuce
Source: Front Plant Sci. 2021 Dec 24;12:781836. doi: 10.3389/fpls.2021.781836 (PMC8739914; doi:10.3389/fpls.2021.781836)

T=Lsat_1_v5_gn_8_45621.1_Q=Lsa−miR1871_S=562

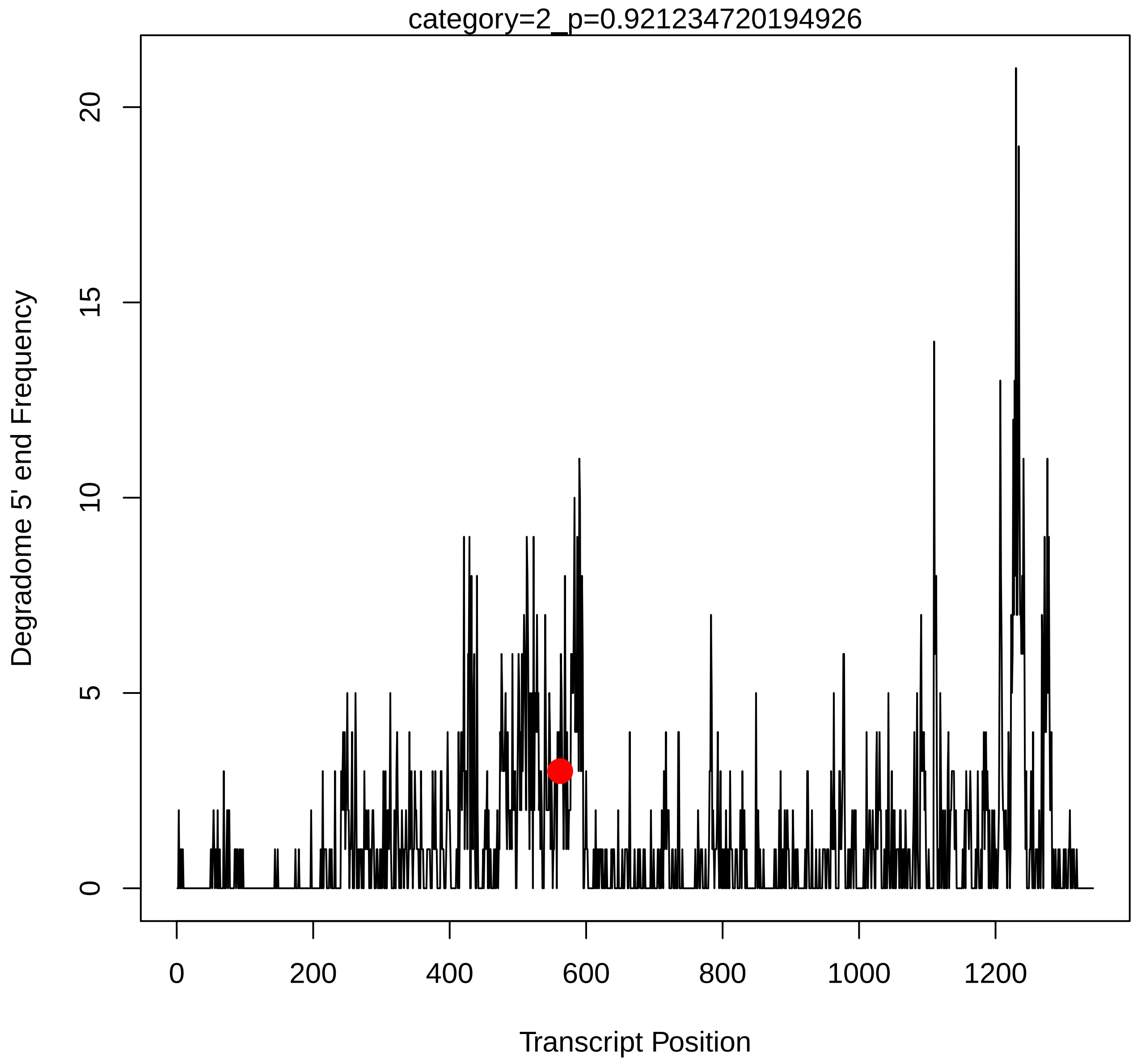

Supplement: Supplementary file 2 [file Data_Sheet_8.ZIP › GSM2230750.plot/Lsa-miR1871_Lsat_1_v5_gn_8_45621.1_562_TPlot.pdf]
